# Predictive Value of Malnutrition, Identified via Different Nutritional Screening or Assessment Tools, for Functional Outcomes in Patients with Stroke: A Systematic Review and Meta-Analysis

**DOI:** 10.3390/nu15143280

**Published:** 2023-07-24

**Authors:** Peiqi Liu, Huimin Tian, Tianliang Ji, Tangsheng Zhong, Lan Gao, Li Chen

**Affiliations:** 1 School of Nursing, Jilin University, Changchun 130021, China; pqliu21@mails.jlu.edu.cn (P.L.); tianhm@jlu.edu.cn (H.T.); zhongtangsheng@jlu.edu.cn (T.Z.); 2 The First Hospital of Jilin University, Changchun 130021, China; tianliang216@jlu.edu.cn; 3 Department of Pharmacology, College of Basic Medical Sciences, Jilin University, Changchun 130021, China

**Keywords:** malnutrition, stroke, outcome, nutritional screening, nutritional assessment

## Abstract

Background: Malnutrition affects more than half of patients with stroke. Although malnutrition leads to more deaths, a longer hospital stay, and higher costs, there is still a lack of consensus regarding the impact of malnutrition on physical functional outcomes in patients with stroke, and there are large differences in the diagnostic effects of nutritional screening or assessment tools for malnutrition. This study aimed to explore the impact of malnutrition in patients with stroke and assess the significance of current nutritional screening and assessment tools for these patients. Methods: Six databases were systematically searched until October 2022. Cohort studies meeting the eligibility criteria were included. Pooled effects were calculated using random-effects models. Results: Twenty-six studies with 21,115 participants were included. The pooled effects of malnutrition on poor functional outcome, FIM points, and dysphagia were OR = 2.72 (95% CI = 1.84–4.06), WMD = −19.42(95% CI = −32.87–−5.96), and OR = 2.80 (95% CI = 1.67–4.67), respectively. Conclusion: Malnutrition adversely affects the recovery of physical and swallowing functions in patients with stroke. Nutritional assessments consistently predict the outcomes of physical function in patients with stroke.

## 1. Introduction

The occurrence of malnutrition poses a burden on patients as well as the health care system [1]. Approximately 19–72% of patients suffer from malnutrition during the course of stroke, and approximately 30% of chronic-phase patients with stroke are left malnourished [2]. The increased malnutrition risk leads to more deaths, a longer hospital stay, increased complications, and higher hospitalization costs in patients with stroke [3,4,5], regardless of the presence of dysphagia [6]. Therefore, it is necessary to pay special attention to nutritional issues in patients with stroke.

The identification of malnutrition serves as the basis for the initiation of active nutritional support [7]. A previous meta-analysis showed that nutrition-related indicators such as body mass index (BMI) and serum albumin levels can predict long-term mortality in patients with stroke [8]. However, the diagnosis of malnutrition using a single biochemical marker has proven unreliable [9], and BMI cannot be used to diagnose malnutrition in obese patients [10]. Different tools should be selected according to the patient population in order to identify malnutrition [11]. In a previous guideline for clinical nutrition in neurology, the Malnutrition Universal Screening Tool (MUST), which is based on the identification of patients with a low BMI, as well as unintentional weight loss and altered nutritional intake due to disease, has been considered appropriate for patients with stroke [12]. In addition, the Controlling Nutritional Status Score (CONUT), Geriatric Nutritional Risk Index (GNRI), Nutritional Risk Screening 2002 (NRS-2002), Subjective Global Assessment (SGA) have been used in patients with stroke for nutritional screening or assessment. These tools are based on a combination of objective measures, such as BMI and weight loss, and hematological measures and subjective measures, including subcutaneous tissue reduction and disease burden. However, there are large differences in the diagnostic effects of the different tools used to measure malnutrition in patients with stroke, and there is no gold standard for the nutritional screening or assessment of these patients [13].

Physical function and quality of life are outcomes that need to be monitored during nutritional therapy [14] and they partly reflect the effectiveness of nutritional therapy and the outcome of stroke. Previous studies [15] have demonstrated the significant impact of malnutrition on the functional outcomes of patients with stroke and several studies have shown that proper nutritional supplementation has a beneficial effect on patients with stroke during the rehabilitation period [16,17,18,19]. However, another meta-analysis demonstrated that the effect of nutritional supplementation on functional outcomes in patients with stroke is not significant [18]. One reason for the inconsistent results may be the lack of consensus on an identification tool for malnutrition in patients with stroke. In addition, several studies have reported that malnutrition affects the recovery of swallowing function in patients with stroke [20,21,22]. In these studies, the sample size was relatively limited and different nutritional screening or assessment tools were used, which may explain the discrepancies in the results of these studies. The aim of our review was to address this knowledge gap by performing a systematic review and meta-analysis of the impact of malnutrition on patients with stroke. We also aim to assess the significance of current nutritional screening and assessment tools for patients with stroke to provide a reference for the clinical nutritional support process.

## 2. Material and Methods

This systematic review and meta-analysis has been registered in PROSPERO (CRD42022379960).

### 2.1. Search Strategy

We systematically searched the following electronic databases: Pubmed, Embase, Cochrane Library, CINAHL, the China national knowledge infrastructure (CNKI), and the Chinese BioMedical Literature Database (CBM) until October 2022. The search strategy consisted of “Malnutrition” or “Nutritional Status”, and “Stroke”, and “outcome”, “Functional Status”, “Deglutition Disorders”, or “Quality of Life”. The full search strategy is listed in the Supplementary Material Text S1. Medical Subject Heading and free-text terms were used in the retrieval process. The reference lists of the previous reviews were manually checked to identify potentially relevant studies.

### 2.2. Eligibility Criteria

Studies meeting the following criteria were considered for inclusion:

*Study design* Cohort studies.

*Participants* Adult (≥18 years old) patients with a primary diagnosis of acute or sub-acute stroke, and not admitted to the intensive care unit.

*Exposure* Patients with malnutrition or nutritional risk determined via nutrition screening or assessment tools at hospital admission within 48 h. Studies that identify malnutrition using a single indicator (e.g., BMI, serum albumin level) or previously unverified tools were excluded.

*Control group* Patients with stroke, and without malnutrition or nutritional risk.

*Outcome* Disability, dysphagia, or quality of life at follow-up period. The measurement of disability included the modified Rankin scale (mRS) or Functional Independence Measure (FIM). Patients with mRS score ≥ 3 were considered to have a disability. Dysphagia is defined as the inability to fully orally intake food for any reason, or a swallowing disorder assessed via any swallowing function assessment tool. The measurement of quality of life included any quantitative tools for quality of life.

### 2.3. Study Selection

Two reviewers searched and reviewed the title and abstract of each study independently. Potentially eligible studies were flagged and the full text was obtained. Two reviewers reviewed the full content of the flagged articles for a final inclusion. A third reviewer supervised the process and resolved any disagreements.

### 2.4. Data Extraction

Data were extracted independently by two authors. Disagreements were discussed and resolved with a third reviewer. The following data were extracted: author, year of publication, country, nutrition screening and assessment tools, time of nutrition screening or assessment, study period, original inclusion criteria, sample size, and main outcomes. Results pertaining to disability, dysphagia, or quality of life in original research were extracted. The odds ratios (ORs) in multivariate analysis were prioritized.

### 2.5. Risk of Bias Assessment

Two reviewers inspected each study to assess the risk of bias independently by using the Risk of Bias In Non-randomized Studies of Interventions (ROBINS-I) tool. The ROBIN-I tool evaluates the following seven domains of bias: bias due to confounding, bias in the selection of participants in the study, bias in classification of interventions, bias due to deviations from intended interventions, bias due to missing data, bias in the measurement of outcomes, and bias in the selection of the reported result. The classification of bias can be either be a “Low”, “Moderate”, “Serious” or “Critical” risk of bias. A third reviewer coordinated the process and resolved any disagreements.

### 2.6. Data Synthesis

Data analyses were implemented using STATA (Release 17; StataCorp LP). Random-effects models were used in the process of effect calculating. The ORs with 95% CI were combined after logarithmic transformation, and the pooled effect sizes were exponentially transformed and expressed as pooled OR and 95% CI. Weighted mean difference (WMD) was used to calculate the pooled effect size of the FIM score between the two groups and expressed as mean and 95% CI. The heterogeneity between studies was assessed using the I^2^ test. Sensitivity analyses were performed to test the stability of the pooled effect sizes by taking turns to exclude the studies. Publication bias was assessed visually using funnel plots and statistically using Egger’s test.

## 3. Results

### 3.1. Study Selection

**Figure 1:**
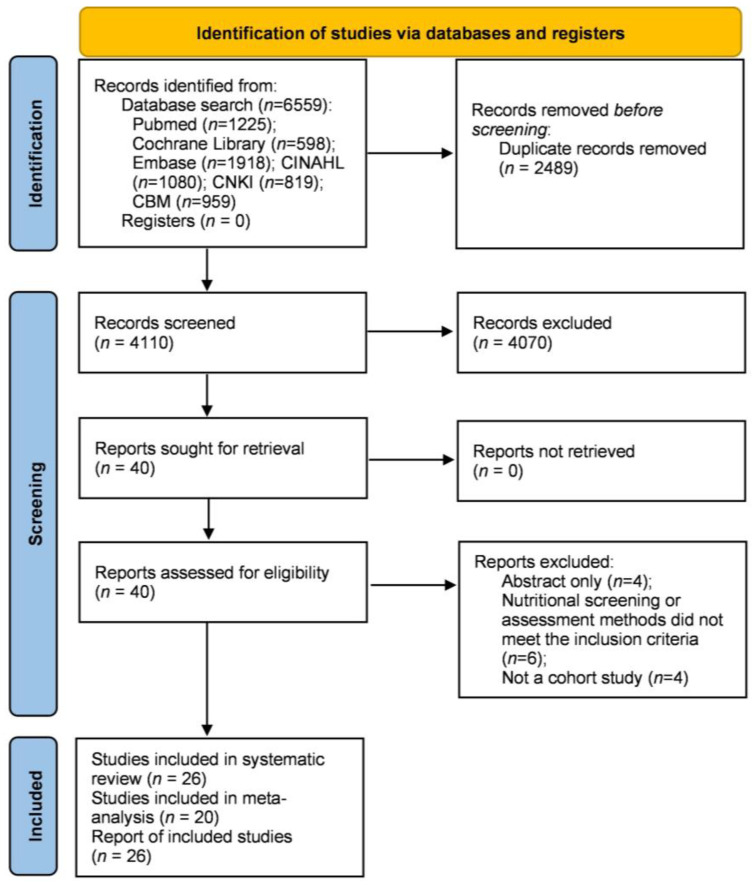
Flow diagram of the literature search process.

The literature search resulted in 6559 studies (Figure 1). After removing duplicates and reviewing titles and abstracts, 40 studies were retrieved for full-text review. Further, 14 studies were excluded for the following reasons: abstract only (*n* = 4), nutritional screening or assessment methods that did not meet the inclusion criteria (*n* = 6), and not a cohort study (*n* = 4). Finally, 26 studies [5,20,21,22,23,24,25,26,27,28,29,30,31,32,33,34,35,36,37,38,39,40,41,42,43,44] were included in this systematic review, 20 of which [5,20,21,22,23,25,26,27,28,29,33,35,37,38,39,40,41,42,43,44] were included for meta-analysis.

### 3.2. Study Characteristics

The characteristics of each study are summarized in Table 1. A total of 21,115 participants from 26 studies were included in this systematic review. Ten studies [23,26,34,35,36,37,38,41,42,43] had prospective designs. The follow-up period of the included studies ranged from the duration of the hospital stay to one year. The average age of participants ranged from 60.38 years to 80.51 years. Most studies were conducted in Asian countries. More than half of studies performed only nutritional screening, not nutritional assessment. Twenty-three studies [20,23,24,25,26,27,28,29,30,31,32,33,34,35,36,37,38,39,40,41,42,43,44] included physical function as an outcome, seventeen [20,23,25,26,27,28,29,33,35,36,37,38,39,40,41,42,43,44] of which were included in the meta-analysis. Five [5,20,21,22,27] studies reported cases of swallowing disorders after the follow-up period and were included in the meta-analysis. Two studies [5,27] reported the impact of malnutrition on the quality of the daily life of patients.

### 3.3. Risk of Bias

Risk of bias was assessed using the ROBINS-I tool. More than half of included studies [20,22,23,24,25,26,31,35,36,37,38,39,40,41,42] were at “moderate” risk of bias, and 11 studies [5,21,27,28,29,30,32,33,34,43,44] were at “serious” risk of bias. The risk of bias mainly arose from confounding factors related to the cohort study design of the included studies. No study was excluded because of a high risk of bias. The risk of bias in the individual studies is presented in Supplementary Material Table S1.

### 3.4. Nutritional Screening and Assessment Method

This review included studies that used various nutritional screening or assessment tools to predict the clinical outcomes of patients with stroke and were conducted in hospital settings when the patient was admitted. This is shown in Table 1. More than half of the studies [22,23,24,26,27,28,30,31,32,33,34,37,38,39,44] used the tools CONUT, GNRI, or PNI. These tools do not require nutrition-related professionals to conduct assessments but directly obtain data from patients or their medical records to calculate risk indices to screen for potential malnourishment. Nutritional screening or assessments were performed by a dietitian or other nutrition-related professional in five studies [5,20,21,25,41], other healthcare providers in two studies [35,40], and a trained investigator in one study [37]. Seventeen studies did not report the professional information of the nutritional screeners or assessors. Five studies [20,21,25,27,37] followed the “two-step” process in order to identify malnutrition, in which the first step used any validated tool to screen and the second step conducted a detailed nutritional assessment for people with potential malnutrition. No studies reported adverse events resulting from nutritional screening or assessment.

### 3.5. Predictive Value of Malnutrition on Function Status after Stroke

**Figure 2:**
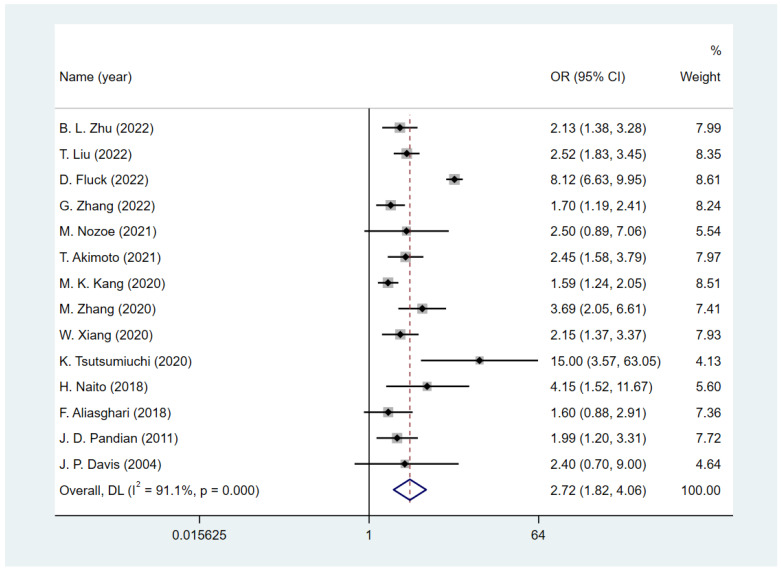
The effect of malnutrition on poor functional outcome in patients with stroke [23,26,28,29,35,36,37,38,39,40,41,42,43,44].

Fourteen studies [23,26,28,29,35,36,37,38,39,40,41,42,43,44] reported the functional status assessed by mRS; a poor functional outcome was defined as mRS ≥ 3, and one study [35] used mRS ≥ 4 as the criterion. The follow-up period of these studies ranged from the duration of the hospital stay to one year. To quantify the predictive value of malnutrition for a poor functional status, we conducted a meta-analysis to calculate the pooled size effect. Adjusted ORs in 11 studies [23,26,28,29,35,36,37,38,39,41,42] and crude ORs in 3 studies [40,43,44] were extracted and pooled. The result (Figure 2) showed that the existing malnutrition screening and assessment tools are good predictors of functional outcome in patients with stroke (OR = 2.72, 95% CI = 1.84 to 4.06; I^2^ = 91.1%).

**Figure 3:**
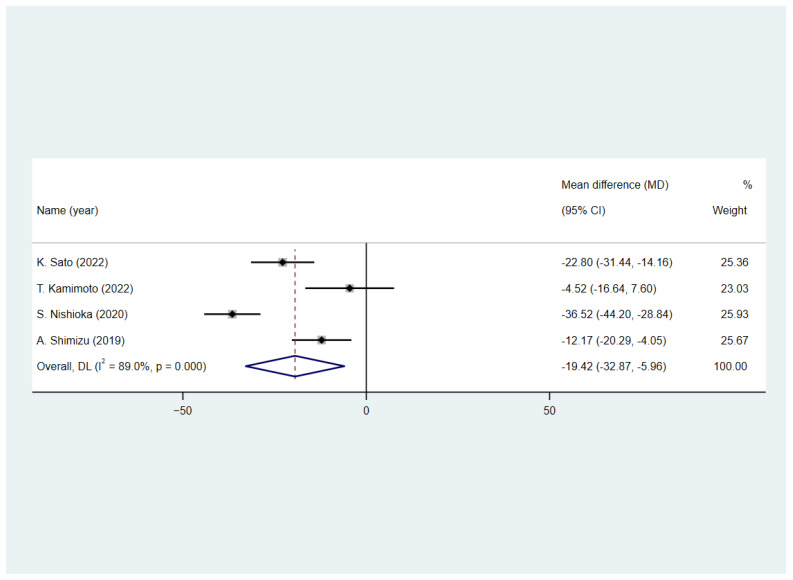
The effect of malnutrition on FIM points in patients with stroke [20,25,27,33].

Figure 3 shows that four studies [20,25,27,33] reported the FIM scores of patients with stroke at hospital discharge. All of them assessed the patients’ FIM scores at discharge. Weighted mean differences (WMDs) were used to calculate the pooled effect size between patients with and without malnutrition. Results (Figure 3) showed that patients with malnutrition have lower FIM scores (WMD = −19.42, 95% CI = −32.87 to −5.96; I^2^ = 89.0%).

Five studies [24,30,31,32,34] did not report follow-up mRS or FIM scores and were not included in the meta-analysis. Results from four studies [24,30,31,34] showed that well-nourished patients improved their FIM scores during hospitalization; however, one study [32] showed that malnutrition had no statistically significant positive effect on the FIM score during hospitalization.

### 3.6. Predictive Value of Malnutrition on Dysphagia

**Figure 4:**
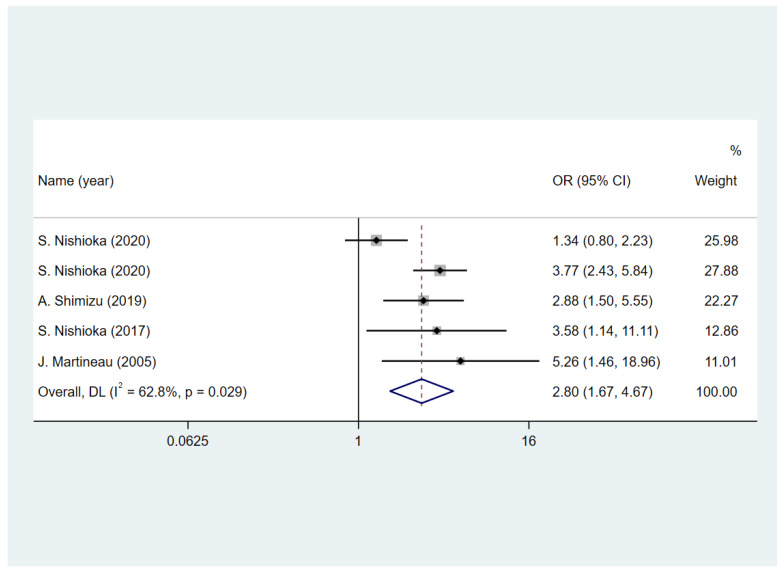
The impact of malnutrition on dysphagia in patients with stroke [5,20,21,22,27].

Five studies [5,20,21,22,27] reported the status of the swallowing function of patients after the follow-up. Among these, four of the studies [5,20,22,27] assessed the status at discharge and one study [21] assessed the status at 6 months. Four studies [5,20,21,22,27] used unadjusted ORs and one [21] presented adjusted hazard ratios. Two studies [20,22] included only patients with dysphagia on admission, whereas the other three [5,21,27] included patients with dysphagia and normal swallowing. The results presented in Figure 4 show that the nutritional assessment and screening tools included in this study have a good predictive effect on swallowing function after follow-up (OR = 2.8, 95% CI = 1.67 to 4.67; I^2^ = 62.8%).

### 3.7. Predictive Value of Malnutrition on Quality of Life

None of the studies using quantitative tools to assess the quality of life met the inclusion criteria. One study [27] showed that patients with stroke and malnutrition were not likely to return to their families after hospital discharge. Another study [5] showed that the impact of malnutrition on patients with stroke returning home was not statistically significant; however, patients with stroke and malnutrition had difficulty returning to a normal diet.

### 3.8. Sensitivity and Subgroup Analyses

The results of the sensitivity analyses showed that the pooled effect sizes of poor functional outcomes were relatively stable (Figure S1). Subgroup analyses were performed according to the nutritional screening or assessment tools used in the included studies. As the vast majority of the included studies were conducted in Asia and most of the participants were patients with ischemic stroke, subgroup analysis by region and stroke type in the protocol was not performed. The results are shown in Table 2 and Table 3, and Supplementary Material Figures S2–S7. Both nutritional screening and assessment tools have a good predictive value for poor functional outcome (OR = 2.29, 95% CI = 1.81 to 2.89; OR = 2.34, 95% CI = 1.84 to 2.99; respectively), and the heterogeneity of the subgroup was relatively low (I^2^ decline from 91.1% to 51.2% and 0%), suggesting that heterogeneity may arise from using different types of tools. Studies using nutritional assessment tools are more precise and less heterogeneous. Malnutrition assessment tools have a good predictive value for lower FIM points (WMD = −21.52, 95% CI = −31.62 to −11.42) compared to the screening tools (WMD = −22.90, 95% CI = −58.16 to 12.36). Both nutritional assessment and screening tools can predict dysphagia (OR = 3.58, 95% CI = 1.15 to 11.17; OR = 2.72, 95% CI = 1.50 to 4.92, respectively).

### 3.9. Publication Bias

The publication bias in the predictive value of malnutrition for poor outcomes was assessed using funnel plots and Egger’s tests. As shown in Supplementary Material Figure S8, no funnel plot asymmetry was found, and publication bias was not statistically significant (*p* = 0.407).

## 4. Discussion

### 4.1. Summary and Interpretation

Nutrition screening or assessment tools that have not been verified in patients with stroke may miss those who are more likely to benefit from nutritional therapy [45]. Therefore, it is essential to assess the ability of each nutritional screening or assessment tool to predict nutrition-related outcomes. Our systematic review included 26 studies with 21,115 cases, aimed at evaluating the effectiveness of current validated tools for nutritional screening and assessment in patients with stroke and at assessing the impact of previous malnutrition on patients with stroke. The results showed that existing nutritional screening and assessment tools have a good predictive value for functional outcomes in patients with stroke. However, the effects and stability of each tool vary between studies.

Several studies have explained the incidence and possible mechanisms of post-stroke malnutrition, indicating that factors such as dysphagia and activity limitation after stroke can lead to a decline in the nutritional status of patients after stroke [46,47,48,49]. However, few studies have focused on the relationship between malnutrition status at the onset of stroke and the impairment of swallowing and mobility. The results of our study, which are consistent with those of previous studies [8,50], suggest that malnutrition upon stroke admission can lead to poor functional status and dysphagia. Our results showed the prognostic value of nutritional status at stroke onset and the longitudinal relationship between malnutrition and the impact of stroke on adverse patient outcomes, indicating that, depending on the conditions of the facilities and human resource availability, it is meaningful to select any one or more of the available nutritional screening or assessment tools for the nutritional screening of stroke patients. In addition, the potential value of aggressive nutritional support, not only in patients with dysphagia, but also in patients with stroke who have been previously diagnosed with malnutrition, should be considered.

Several studies [22,23,24,26,27,28,30,31,32,33,34,37,38,39,44] have used the CONUT and GNRI as screening tools for malnutrition. The CONUT score is based on the serum albumin level, total cholesterol level, lymphocyte count, design for detection, and continuous control of hospital undernutrition [51]. The GNRI is used to predict clinical outcomes in elderly patients and is based on body weight, height, and albumin level [52]. Both tools are based on objective indicators and include the albumin level as a factor that is generally believed to be related to the nutritional status of patients [53]. However, CONUT focuses on reflecting the nutritional status, while GNRI focuses on predicting the poor prognosis caused by nutritional problems. The CONUT and GNRI have been validated in patients with cancer [54,55], heart failure [56,57], and elderly adults [58]. Our results showed that the CONUT and GNRI have also been properly validated for predicting functional prognosis in patients with stroke, indicating the potential benefits of the CONUT and GNRI in clinical settings or in communities that lack nutritionists. CONUT showed a higher heterogeneity in our meta-analysis, and the predictive value of the FIM score was not statistically significant. This may be because the GNRI was designed for elderly people [55], which resulted in a better agreement between the study populations. In additioon, it should be noted that CONUT and GNRI are partially dependent on serum albumin levels, which have been shown in previous studies to be susceptible to inflammation and humoral levels [59,60], which may limit the effect of COUNT and GNRI in patients with inflammation.

The NRS-2002 tool was developed to screen patients who may benefit from nutritional support. It is combined with an impaired nutritional status score, severity of disease score, and age score [61]. As one of the most popular malnutrition screening tools [62], the NRS-2002 is recommended by ESPEN as a screening tool for malnutrition in hospitalized patients [63] and has moderate validity and agreement in many settings [11]. Factors such as low BMI, unintentional weight loss, reduction in food intake, and disease severity have been widely validated over the past few decades and are included in multiple nutritional screening and assessment tools [7]. In a recent study, an NRS-2002 score of ≥3 points was confirmed as an independent risk factor for stroke-associated infections [64]; these results reflect the potential value of the NRS-2002 tool to identify nutrition-related risks in patients with stroke. Our review showed that the NRS-2002 score has a good predictive value for physical function outcomes in patients with stroke, which means that NRS-2002 may be a viable nutritional screening tool to be used at the beginning of the rehabilitation process in order to provide nutritional support.

The tool MUST is based on the identification of patients with a low BMI, unintentional weight loss, and an altered nutritional intake due to disease. It has been developed for adults in all healthcare settings [65] and has been recently validated in patients with stroke for predicting death, disability, infections, and length of hospital stay [66]. The results of a study that included 1146 outpatients and hospitalized patients showed that the MUST score was better correlated with the ESPEN criteria for the definition of malnutrition than the score obtained using the NRS-2002 tool [62]. Our results also demonstrate the great potential value of MUST in screening for malnutrition in patients with stroke. In the latest guidelines for clinical nutrition in neurology proposed by ESPEN, the MUST tool is recommended for screening the risk of malnutrition and identifying patients who may benefit from nutritional support [12]; however, further research is still needed to provide more evidence of the value of the MUST tool for nutritional screening in patients with stroke.

Nutritional assessment should be performed for every patient who is at risk after nutritional screening [14,67]. According to ESPEN, predefined tools, such as SGA [68], PG-SGA [69], and MNA [70], can be used for nutritional assessment [14]. In recent years, diagnostic criteria for malnutrition, such as ESPEN-DCM [71] and GLIM criteria [7], have also provided a process for nutritional assessment. Although some of these tools are considered as the gold or semi-gold standards for assessing malnutrition [72], few studies have evaluated the predictive effects of these criteria on functional outcomes in patients with stroke. One possible reason is that the assessment of nutritional status requires nutrition-related professionals to obtain comprehensive and detailed information with respect to patients’ nutritional conditions in order to formulate a diagnosis. However, the process of malnutrition screening can be handled by any healthcare provider [73]. These tools include reduced food intake, disease burden, weight loss, and body mass as assessment factors. The factors in the SGA tool also include fat mass, fluid retention, and muscle function, making SGA the most comprehensive evaluation tool. However, the SGA tool relies on subjective judgment and its accuracy depends on the experience of the assessor [74], which may be one of the reasons limiting its wide application. The ESPEN-DCM is a new diagnostic tool for assessing malnutrition based on evidence and expert consensus. Weight loss combined with a low BMI or Fat-Free Mass Index (FFMI) is used to diagnose malnutrition [71], making it a diagnostic criterion for malnutrition based primarily on objective indicators. The GLIM criteria also assess malnutrition status and are mainly based on objective indicators. The criteria for diagnosing malnutrition include weight loss, low BMI, and reduced muscle mass as phenotypic criteria; reduced food intake or assimilation and inflammation as etiologic criteria; and the combination of both phenotypic and etiologic criteria [7]. Both the GLIM criteria and ESPEN-DCM have been established through expert consensus; therefore, their diagnostic and prognostic effects on malnutrition need to be verified in various populations. In addition, these two criteria use the “two-step” diagnosis of malnutrition; the first step is to screen the patients at risk, and the second step is to assess the nutritional status for malnutrition diagnosis [7,71]. A previous study showed that the selection of screening tools affects the final diagnostic effect, and thus appropriate screening tools should be selected in different populations [75]; further studies are needed to evaluate the effects of combinations of different screening tools and assessment tools in patients with stroke. In general, our analysis showed that most of the included nutritional assessment tools had the ability to predict physical functional outcomes, and lower heterogeneity was found across studies, suggesting the greater stability of assessment tools relative to screening tools. Moreover, the predictive effect of the ESPEN-DCM, GLIM criteria, and PG-SGA tools on swallowing function has also been verified in a few studies. These results are partly consistent with those of previous studies [8], further emphasizing the significance of nutritional assessment and the impact of malnutrition at stroke onset on the outcomes of patients with stroke.

### 4.2. Strengths

Our systematic review and meta-analysis pooled the results of 26 studies. To the best of our knowledge, this is the first systematic review focusing on the impact of malnutrition, identified using current screening or assessment tools, on physical function, swallowing function, and the quality of life of patients with stroke. The included studies had a longitudinal design, which further emphasized the impact of preexisting malnutrition on clinical outcomes, as well as the significance of screening and evaluation in patients with stroke.

### 4.3. Limitations

Our study had several limitations. Due to the lack of original studies, no meta-analysis of the quality of life was possible. Meanwhile, a subgroup analysis was not conducted according to different research designs, regions, disease periods, hospital grades, or other factors to explain the reasons for the heterogeneity in our meta-analysis. Some studies had a retrospective design, which may have led to a relatively high risk of bias. Most original studies did not consider the nutritional support received by patients during hospitalization, which could have led to more confounding factors.

### 4.4. Implications

In our systematic review and meta-analysis, we quantitatively summarized the impact of malnutrition on the recovery of physical and swallowing functions in patients with stroke, and clarified the importance of nutritional screening and assessment; we also clarified the significant impact of malnutrition on patients with stroke. During the process of subgroup analysis, we found that one of the reasons for the heterogeneity among different studies is the differences in the nutritional screening or assessment tools used. Further studies are needed to evaluate their value in screening for malnutrition in patients with stroke and their effectiveness in working with diagnostic tools for malnutrition, such as GLIM criteria and ESPEN-DCM. In addition, our review showed the synergistic effects of malnutrition and stroke on the functional recovery of patients with stroke, suggesting that healthcare providers should focus on malnutrition not only for patients with dysphagia or unconsciousness, but also for all patients at an earlier stage of stroke onset. Healthcare providers should also provide nutritional assessment and nutritional intervention to patients at risk, and evaluate the effect of nutritional intervention.

## 5. Conclusions

Malnutrition at admission has adverse effects on the recovery of physical and swallowing functions in patients with stroke. Most of the nutritional screening and assessment tools included in our review have a good predictive value for functional outcomes in patients with stroke, but the predicted values of different tools varied. Nutritional assessments were more consistently predictive of the outcomes of physical function in patients with stroke. Higher heterogeneity was observed among nutritional screening tools. Further research is needed to demonstrate the impact of malnutrition on the long-term quality of life of patients with stroke and to clarify the potential value of current malnutrition screening and assessment tools in the process of nutritional therapy.

## Figures and Tables

**Table 1:** Characteristics of included studies.

Author, Year Published	Country	Study Design	Study Population	Sample Size (*n*)	Age (Mean ± SD, Years)	Gender (*n*, Female/Male)	Screening or Assessment Tools	Follow-Up Time	Outcome
B. L. Zhu, 2022 [23]	China	prospectively	patients with hemorrhagic stroke	328	60.38 ± 12.38	109/219	NRS2002, COUNT	after 3 months	physical functional status
K. Sato, 2022 [25]	Japan	retrospective	elderly patients with subacute stroke	183	79.7 ± 7.5	80/103	GLIM criteria	at hospital discharge	physical functional status
T. Liu, 2022 [29]	China	retrospective	patients with dysphagia after acute stroke	789	Not mentioned	261/528	NRS2002, SGA	after 3 months	physical functional status
E. C. Lee, 2022 [30]	South Korea	retrospective	patients with stroke	117	67.77 ± 15.15	54/63	COUNT	after 1 month	physical functional status
T. Kamimoto, 2022 [33]	Japan	retrospective	elderly patients with subacute stroke	205	77.8 ± 7.1	107/98	COUNT	at hospital discharge	physical functional status
D. Fluck, 2022 [35]	UK.	prospectively	patients with stroke	2962	73.5 ± 13.1	1447/1515	MUST	at hospital discharge	physical functional status
G. Zhang, 2022 [38]	China	prospectively	patients with acute ischemic stroke	8698	62.26 ± 11.25	2706/5992	GNRI, COUNT, PNI	after 12 months	physical functional status
M. Nozoe, 2021 [26]	Japan	prospectively	elderly patients with acute stroke	324	76 ± 11	137/187	GNRI	after 3 months	physical functional status
T. Akimoto, 2021 [44]	Japan	retrospective	elderly patients with acute ischemic stroke	218	80.51 ± 25.37	81/137	COUNT, GNRI	at hospital discharge	physical functional status
D. Scrutinio, 2020 [24]	Italy	retrospective	patients with subacute ischemic stroke	668	75 (67–81)	325/363	PNI	at hospital discharge	physical functional status
S. Nishioka, 2020 [21]	Japan	retrospective	patients over 50 years old with dysphagia after acute stroke	113	77 (66–83)	58/55	ESPEN-DCM	after 6 months	dysphagia
S. Nishioka, 2020 [27]	Japan	retrospective	elderly patients with subacute stroke	420	78.1 ± 7.9	171/249	MNA-SF, GNRI, ESPEN-DCM	at hospital discharge	physical functional status, dysphagia, quality of life
Y. Kokura, 2020 [32]	Japan	retrospective	elderly patients with stroke	702	76.3 ± 12	334/368	COUNT	at hospital discharge	physical functional status
H. Irisawa, 2020 [34]	Japan	prospectively	patients with subacute stroke	179	79.5 ± 11.5	90/89	GNRI	after 1 month	physical functional status
M. K. Kang, 2020 [42]	South Korea	prospectively	patients with stroke	1906	67.77 ± 12.30	738/1168	GNRI	after 3 months	physical functional status
M. Zhang, 2020 [37]	China	prospectively	patients with stroke	593	67.3 ± 12.0	237/356	COUNT, GNRI, MUST, NRS-2002, ESPEN-DCM	after 3 months	physical functional status
W. Xiang, 2020 [39]	China	retrospective	patients after thrombolytic therapy	405	66 ± 16	210/195	COUNT, PNI	after 3 months	physical functional status
K. Tsutsumiuchi, 2020 [40]	Japan	retrospective	patients with subacute stroke and functional impairment	90	75 ± 8.7	43/47	MNA-SF	at hospital discharge	physical functional status
A. Shimizu, 2019 [20]	Japan	retrospective	elderly patients with dysphagia after acute stroke	188	78.9 ± 7.7	68/120	GLIM criteria	at hospital discharge	physical functional status, dysphagia
H. Naito, 2018 [28]	Japan	retrospective	patients with acute ischemic stroke	264	70.9 ± 12.2	93/171	COUNT	after 3 months	physical functional status
F. Aliasghari, 2018 [43]	Iran	prospectively	patients with ischemic stroke	253	74.42 ± 7.8	120/133	MNA	after 3 months	physical functional status
S. Nishioka, 2017 [22]	Japan	retrospective	patients with dysphagia after stroke	264	78.5 ± 7.5	109/155	GNRI	at hospital discharge	dysphagia
Y. Kokura, 2016 [31]	Japan	retrospective	patients with subacute stroke	540	80 (75–85)	269/271	GNRI	at hospital discharge	physical functional status
J. D. Pandian, 2011 [41]	India	prospectively	patients with stroke	448	58.66 ± 13.7	110/216	SGA	after 1 month	physical functional status
J. Martineau, 2005 [5]	Australia	retrospective	patients with stroke	73	72.78 ± 12.98	-	PG-SGA	at hospital discharge	dysphagia, quality of life
J. P. Davis, 2004 [36]	Australia	prospectively	patients with stroke	185	Not mentioned	87/98	SGA	after 1 month	physical functional status

Abbreviations: NRS-2002: Nutritional Risk Screening 2002; COUNT: Controlling Nutritional Status Score; GLIM criteria: Global Leadership Initiative on Malnutrition—Criteria for the Diagnosis of Malnutrition; SGA: Subjective Global Assessment; MUST: Malnutrition Universal Screening Tool; GNRI: Geriatric Nutritional Risk Index; PNI: Prognostic Nutritional Index; ESPEN-DCM: European Society of Parenteral and Enteral Nutrition—Diagnostic Criteria for Malnutrition; MNA-SF: Mini Nutritional Assessment—Short Form; PG-SGA: Patient-Generated Subjective Global Assessment.

**Table 2:** Subgroup analysis of nutritional screening tools or assessment tools.

	Number of Studies (*n*)	Poor Functional Outcome (OR with 95% CI)	I^2^	Number of Studies (*n*)	FIM Scores (WMD with 95% CI)	I^2^	Number of Studies (*n*)	Dysphagia (OR with 95% CI)	I^2^
Nutritional screening tools	10	2.29 (1.81 to 2.89)	51.2%	2	−22.90 (−58.16 to 12.36)	96.3%	1	3.58 (1.15 to 11.17)	0%
Nutritional assessment tools	5	2.34 (1.84 to 2.99)	0%	3	−21.52 (−31.62 to −11.42)	78.3%	4	2.72 (1.50 to 4.92)	71.40%

**Table 3:** Subgroup analysis of different screening or assessment tools.

Tools	Number of Studies (*n*)	Poor Functional Outcome (OR with 95% CI)	I^2^	Number of Studies (*n*)	FIM Scores (WMD with 95% CI)	I^2^	Number of Studies (*n*)	Dysphagia (OR with 95% CI)	I^2^
COUNT	6	2.04 (1.44 to 2.88)	72.1%	1	−4.52 (−16.64 to 7.60)	0%	-	-	-
GNRI	5	1.67 (1.31 to 2.13)	52.4%	1	−41.73 (−50.48 to −32.97)	0%	1	3.58 (1.15 to 11.17)	0%
SGA	3	2.32 (1.69 to 3.19)	0%	-	-	-	-	-	-
NRS2002	3	2.69 (1.74 to 4.15)	40.8%	-	-	-	-	-	-
MUST	2	8.33 (6.88 to 10.08)	0.0%	-	-	-	-	-	-
PNI	2	3.00 (2.11 to 4.27)	13.0%	-	-	-	-	-	-
MNA	1	1.60 (0.88 to 2.91)	0%	-	-	-	-	-	-
MNA-SF	1	15.00 (3.57 to 63.04)	0%	1	−39.21 (−48.22 to −30.21)	0%	-	-	-
ESPEN-DCM	1	3.05 (1.64 to 5.65)	0%	1	−29.43(−37.13 to −21.74)	0%	2	2.27 (0.82 to 6.25)	89.0%
GLIM criteria	-	-	-	2	–17.38 (−27.79 to−6.97)	67.6%	1	2.88 (1.50 to 5.55)	0%
PG-SGA	-	-	-	-	-	-	1	5.26 (1.46 to 18.96)	0%

## Data Availability

Data sharing not applicable. No new data were created or analyzed in this study. Data sharing is not applicable to this article.

## Supplementary Materials

The following supporting information can be downloaded at: https://www.mdpi.com/article/10.3390/nu15143280/s1. Text S1: Search strategy. Figure S1: Sensitivity analyses of the effect of malnutrition on poor outcome in patients with stroke. Figures S2–S7: Subgroup analysis of outcomes. Figure S8: Funnel plot. Table S1: Risk of bias assessment of NRSIs by ROBIN-I tool.

## Abbreviations

BMI, body mass index; CONUT, Controlling Nutritional Status Score; ESPEN-DCM, European Society of Parenteral and Enteral Nutrition—Diagnostic Criteria for Malnutrition; GLIM criteria, Global Leadership Initiative on Malnutrition—Criteria for the Diagnosis of Malnutrition; GNRI, Geriatric Nutritional Risk Index; MNA, Mini Nutritional Assessment; mRS, modified Rankin scale; MUST, Malnutrition Universal Screening Tool; NRS-2002, Nutritional Risk Screening 2002; OR, odds ratio; PG-SGA, Patient-Generated Subjective Global Assessment; PNI, Prognostic Nutritional Index; SGA, Subjective Global Assessment; WMD, weighted mean difference.
