# Supplementary material for: Predictive Value of Malnutrition, Identified via Different Nutritional Screening or Assessment Tools, for Functional Outcomes in Patients with Stroke: A Systematic Review and Meta-Analysis
Source: Nutrients. 2023 Jul 24;15(14):3280. doi: 10.3390/nu15143280 (PMC10383200; doi:10.3390/nu15143280)
Supplement: Supplementary file 1 [file nutrients-15-03280-s001.zip › nutrients-2498467-supplementary.pdf]

Supplementary material to the manuscript

Predictive value of malnutrition identified by different nutritional screening or assessment tools on functional outcome in patients with stroke: A systematic review.

Text S1: Search strategy

Figure S1: Sensitivity analyses of the effect of malnutrition on poor outcome in patients with stroke
Figures S2–S7: Subgroup analysis of outcomes
Figure S8: Funnel plot

Table S1: Risk of bias assessment of NRSIs by ROBIN-I tool.

Text S1. Search strategy

Pubmed

#1 (Malnutrition[MeSH Terms]) OR (Malnutriti*[Title/Abstract]) OR (Nutritional Deficienc*[Title/Abstract]) OR (Undernutriti*[Title/Abstract]) OR (Malnourishmen*[Title/Abstract]) OR (Nutritional Status[MeSH Terms]) OR (Nutriti* Status[Title/Abstract]) OR (nutriti*[Title/Abstract])

#2 (stroke[MeSH Terms]) OR (Strok*[Title/Abstract]) OR (Cerebrovascular Acciden*[Title/Abstract]) OR (CVA[Title/Abstract]) OR (Apoplexy[Title/Abstract]) OR (Brain Vascular Acciden*[Title/Abstract])

#3 (Deglutition Disorders[MeSH Terms]) OR (Deglutition Disorde*[Title/Abstract]) OR (Swallowing Disorde*[Title/Abstract]) OR (Dysphagia[Title/Abstract]) OR (Quality of Life[MeSH Terms]) OR (Quality of Life[Title/Abstract]) OR (Life Quality[Title/Abstract]) OR (HRQOL[Title/Abstract]) OR (QOL[Title/Abstract]) OR (Recovery of Function[MeSH Terms]) OR (Recove* Function[Title/Abstract]) OR (Function Recove*[Title/Abstract]) OR (Activities of Daily Living[MeSH Terms]) OR (ADL[Title/Abstract]) OR (Daily Living Activi*[Title/Abstract]) OR (Chronic Limitation Activi*[Title/Abstract]) OR (Functional Status[MeSH Terms]) OR (Functional Independence[Title/Abstract]) OR (Functional Dependence[Title/Abstract]) OR (physical function[Title/Abstract]) OR (disability[Title/Abstract]) OR (outcome[Title/Abstract])

#4 #1 AND #2 AND #3

Embase

#1 (malnutriti*:ti,ab,kw OR (nutritional:ti,ab,kw AND deficienc*:ti,ab,kw) OR undernutriti*:ti,ab,kw OR malnourishmen*:ti,ab,kw OR (nutriti*:ti,ab,kw AND status:ti,ab,kw) OR nutriti*:ti,ab,kw) AND [embase]/lim

#2 (strok*:ti,ab,kw OR (cerebrovascular:ti,ab,kw AND acciden*:ti,ab,kw) OR cva:ti,ab,kw OR apoplexy:ti,ab,kw OR (brain:ti,ab,kw AND vascular:ti,ab,kw AND acciden*:ti,ab,kw)) AND [embase]/lim

#3 (deglutition:ti,ab,kw AND disorde*:ti,ab,kw OR (swallowing:ti,ab,kw AND disorde*:ti,ab,kw) OR dysphagia:ti,ab,kw OR (quality:ti,ab,kw AND of:ti,ab,kw AND life:ti,ab,kw) OR (life:ti,ab,kw AND quality:ti,ab,kw) OR hrqol:ti,ab,kw OR qol:ti,ab,kw OR (recove*:ti,ab,kw AND of:ti,ab,kw AND function:ti,ab,kw) OR (function:ti,ab,kw AND recove*:ti,ab,kw) OR adl:ti,ab,kw OR (daily:ti,ab,kw AND living:ti,ab,kw AND activi*:ti,ab,kw) OR (chronic:ti,ab,kw AND limitation:ti,ab,kw AND of:ti,ab,kw AND activi*:ti,ab,kw) OR (functional:ti,ab,kw AND independence:ti,ab,kw) OR (functional:ti,ab,kw AND dependence:ti,ab,kw) OR (physical:ti,ab,kw AND function:ti,ab,kw)) OR (disability:ti,ab,kw) OR (outcome:ti,ab,kw) AND [embase]/lim

#4 #1 AND #2 AND #3

CINAHL
S1 (MM "Malnutrition+") OR Malnutriti* OR Nutritional Deficienc* OR Undernutriti* OR Malnourishmen*
S2 (MM "Nutritional Status+") OR Nutriti* Status OR Nutriti*
S3 (MM "stroke+") OR Strok* OR Cerebrovascular Acciden* OR CVA OR Apoplexy OR Brain Vascular Acciden*
S4 (MM "Deglutition Disorders+") OR Deglutition Disorde* OR Swallowing Disorde* OR Dysphagia
S5 (MM "Quality of Life+") OR Quality of Life OR Life Quality OR HRQOL OR QOL
S6 (MM "Recovery of Function+") OR Recove* of Function OR Function Recove*
S7 (MM "Activities of Daily Living+") OR ADL OR Daily Living Activi* OR Chronic Limitation of Activi*
S8 (MM "Functional Status+") OR Functional Independence OR Functional Dependence OR physical function OR disability or outcome
S9 (S1 OR S2) AND S3 AND (S4 OR S5 OR S6 OR S7 OR S8)

Cochrane Library
#1 MeSH descriptor: [Malnutrition] explode all trees
#2 (Malnutriti*):ti,ab,kw OR (Nutritional Deficienc*):ti,ab,kw OR (Undernutriti*):ti,ab,kw OR (Malnourishmen*):ti,ab,kw
#3 MeSH descriptor: [Nutritional Status] explode all trees
#4 (Nutriti* Status):ti,ab,kw OR (Nutriti*):ti,ab,kw
#5 MeSH descriptor: [Stroke] explode all trees
#6 (Strok*):ti,ab,kw OR (Cerebrovascular Acciden*):ti,ab,kw OR (CVA):ti,ab,kw OR (Apoplexy):ti,ab,kw OR (Brain Vascular Acciden*):ti,ab,kw
#7 MeSH descriptor: [Deglutition Disorders] explode all trees
#8 (Deglutition Disorde*):ti,ab,kw OR (Swallowing Disorde*):ti,ab,kw OR (Dysphagia):ti,ab,kw
#9 MeSH descriptor: [Quality of Life] explode all trees
#10 (Quality of Life):ti,ab,kw OR (Life Quality):ti,ab,kw OR (HRQOL):ti,ab,kw OR (QOL):ti,ab,kw
#11 MeSH descriptor: [Recovery of Function] explode all trees
#12 (Recove* of Function):ti,ab,kw OR (Function Recove*):ti,ab,kw
#13 MeSH descriptor: [Activities of Daily Living] explode all trees
#14 (ADL):ti,ab,kw OR (Daily Living Activi*):ti,ab,kw OR (Chronic Limitation of Activi*):ti,ab,kw
#15 MeSH descriptor: [Functional Status] explode all trees
#16 (Functional Independence):ti,ab,kw OR (Functional Dependence):ti,ab,kw OR (physical function):ti,ab,kw OR (disability):ti,ab,kw OR (outcome):ti,ab,kw
#17 (#1 OR #2 OR #3 OR #4) AND (#5 OR #6) AND (#7 OR #8 OR #9 OR #10 OR #11 OR #12 OR #13 OR #14 OR #15 OR #16)

CNKI

(TI=('营养不良'+'营养不足'+'营养状态') OR AB=('营养不良'+'营养不足'+'营养状态')) AND (TI=('中风'+'脑出血'+'脑梗'+'脑血管意外'+'卒中'+'脑缺血') OR AB=('中风'+'脑出血'+'脑梗'+'脑血管意外'+'卒中'+'脑缺血')) AND (TI=('吞咽障碍'+'吞咽功能'+'吞咽困难'+'生活质量'+'QOL'+'身体功能'+'活动能力'+'功能恢复'+'ADL'+'生活能力'+'功能状态'+'自理'+'残疾+'结局') OR AB=('吞咽障碍'+'吞咽功能' +'生活质量'+'QOL'+'身体功能'+'活动能力'+'功能恢复'+'ADL'+'生活能力'+'功能状态'+'自理'+'残疾'+'结局'))

CBM

1 "营养不良"[不加权:扩展] OR "营养状态" OR "营养不足" OR "营养不良"

2 "卒中"[不加权:扩展] OR "卒中" OR "缺血性脑卒中" OR "出血性脑卒中" OR "脑梗死"

3 "吞咽障碍"[不加权:扩展] OR "吞咽障碍" OR "吞咽困难" OR "生活质量"[不加权:扩展] OR "生活质量" OR "QOL" OR "日常生活活动"[不加权:扩展] OR "ADL" OR "功能恢复"[不加权:扩展] OR "功能恢复" OR "功能状态"[不加权:扩展] OR "功能状态" OR "身体功能" OR "残疾"[不加权:扩展] OR "残疾" OR "结局"[不加权:扩展] OR "结局"

4 1 AND 2 AND 3

Figure S1. Sensitivity analyses of the effect of malnutrition on poor outcome in patients with stroke

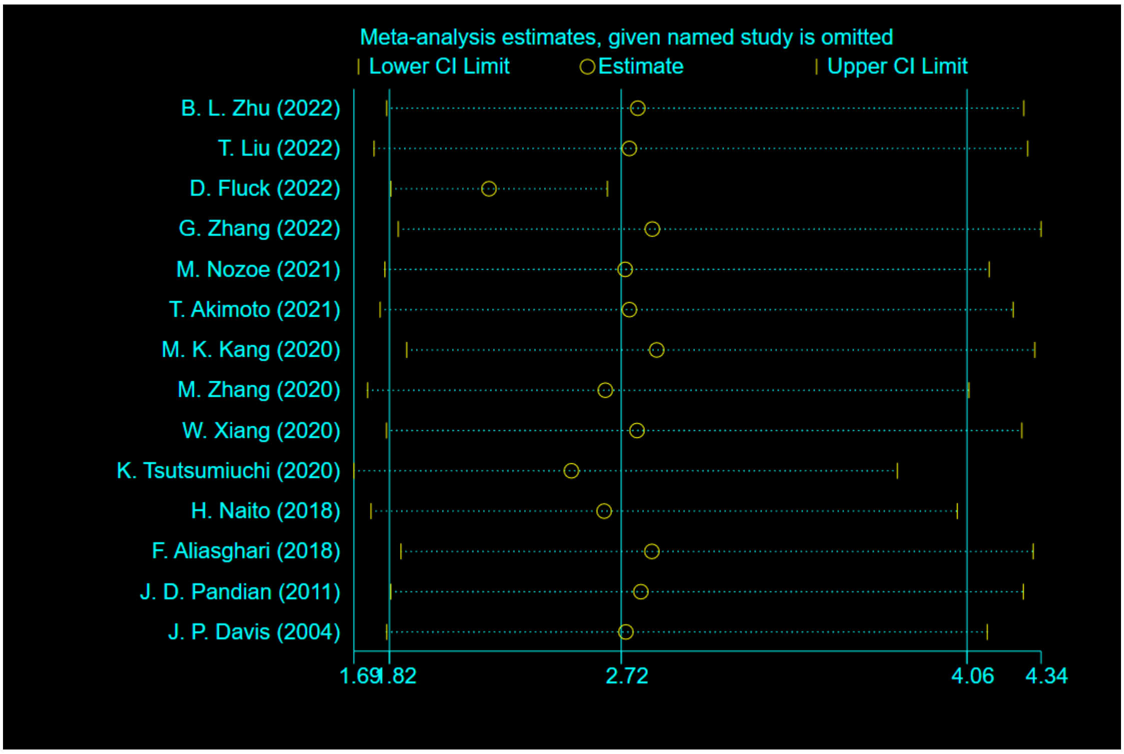

Figure S2. Subgroup analysis of the impact of malnutrition identified by screening or assessment on poor functional outcomes in patients with stroke.

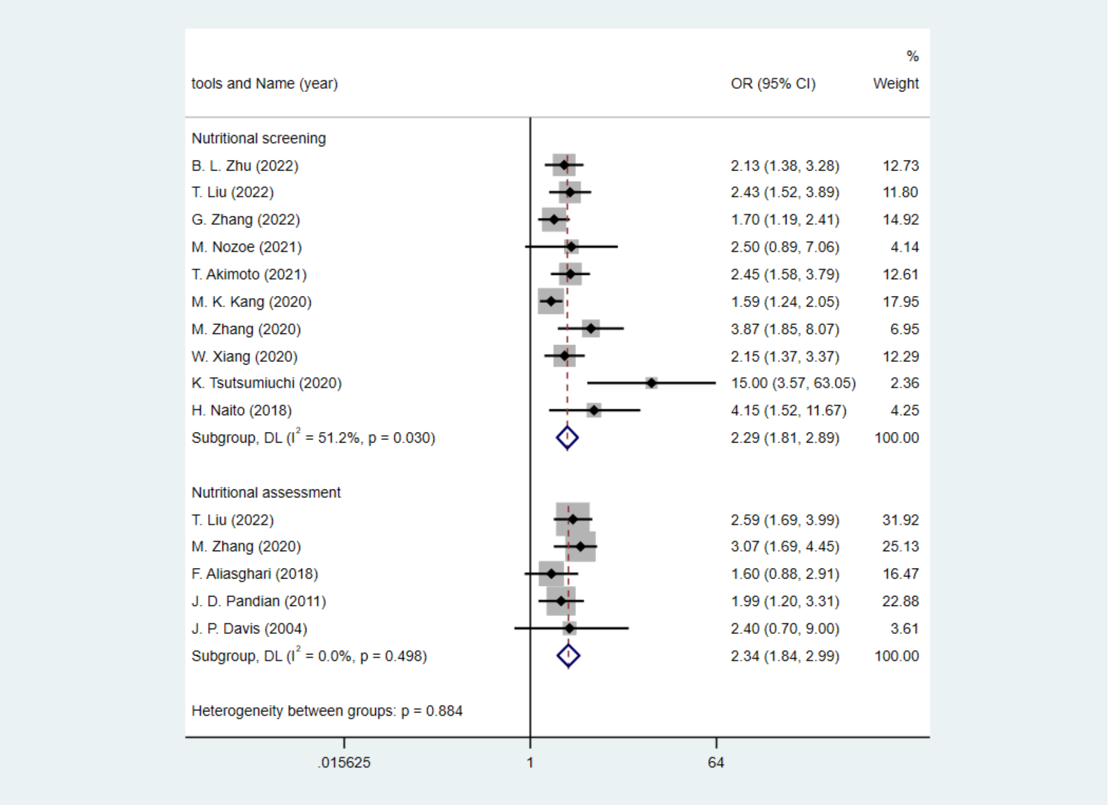

Figure S3. Subgroup analysis of the impact of malnutrition identified by screening or assessment on FIM points in patients with stroke.

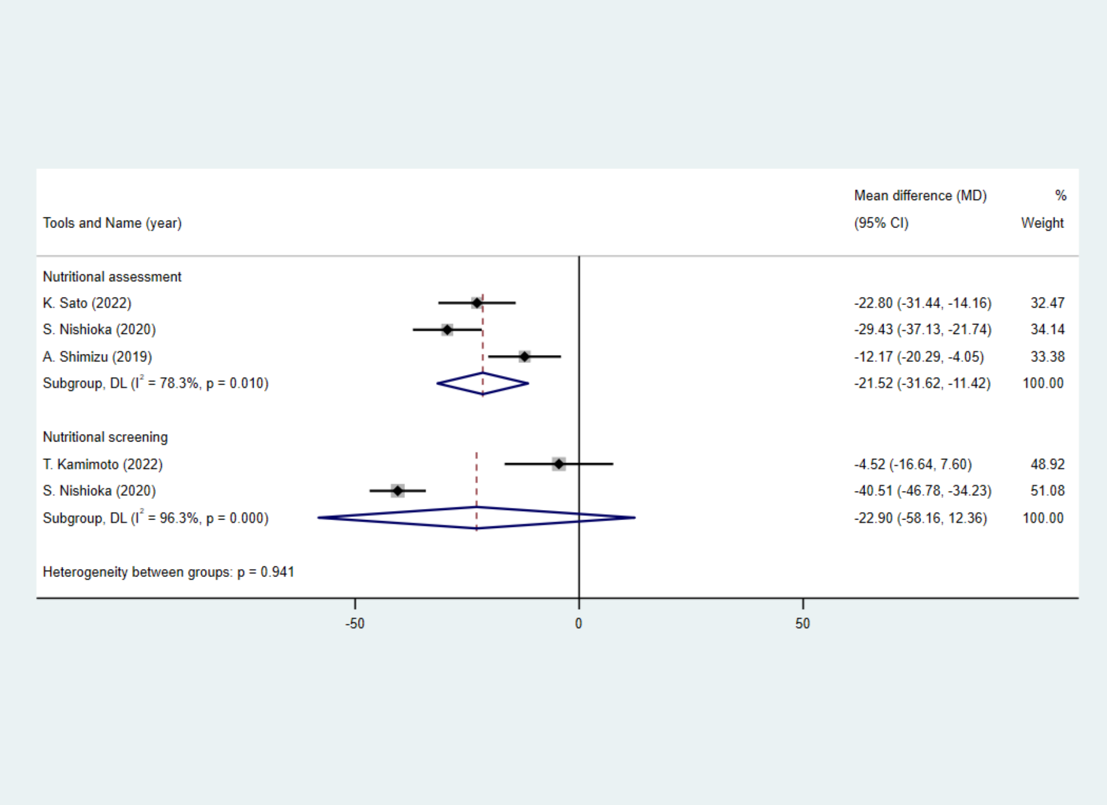

Figure S4. Subgroup analysis of the impact of malnutrition identified by each tool on FIM points in patients with stroke.

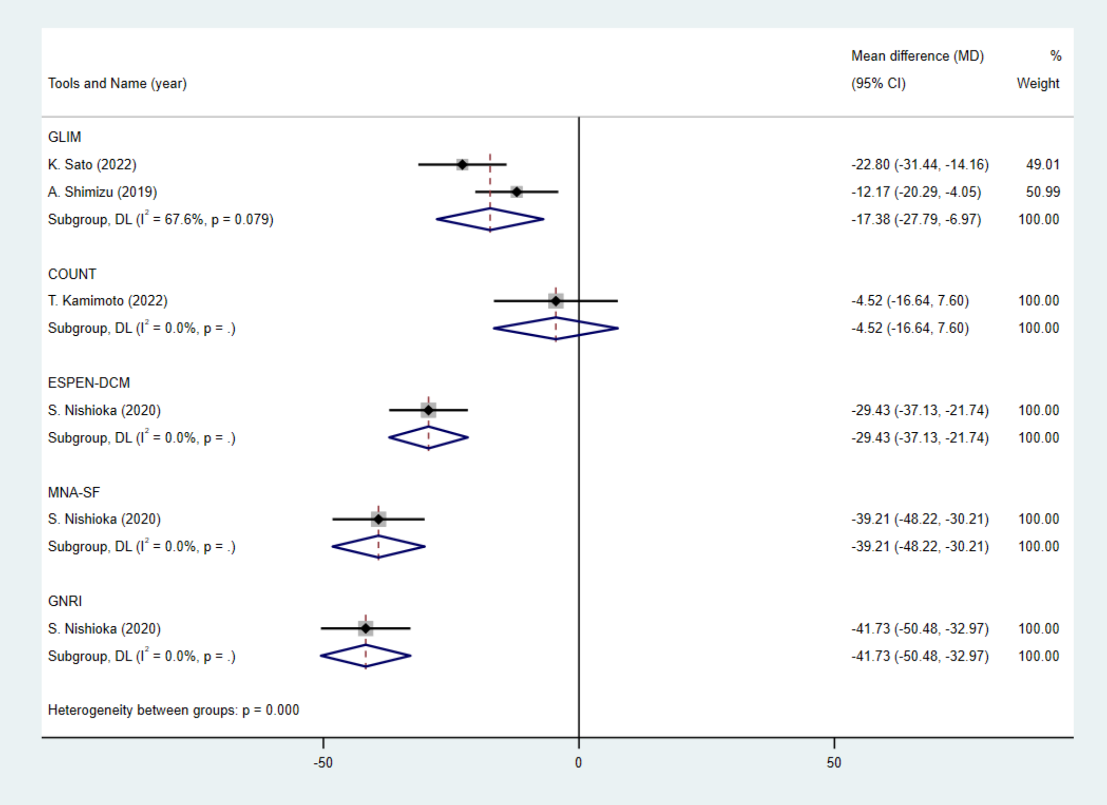

Figure S5. Subgroup analysis of the impact of malnutrition identified by screening or assessment on dysphagia in patients with stroke.

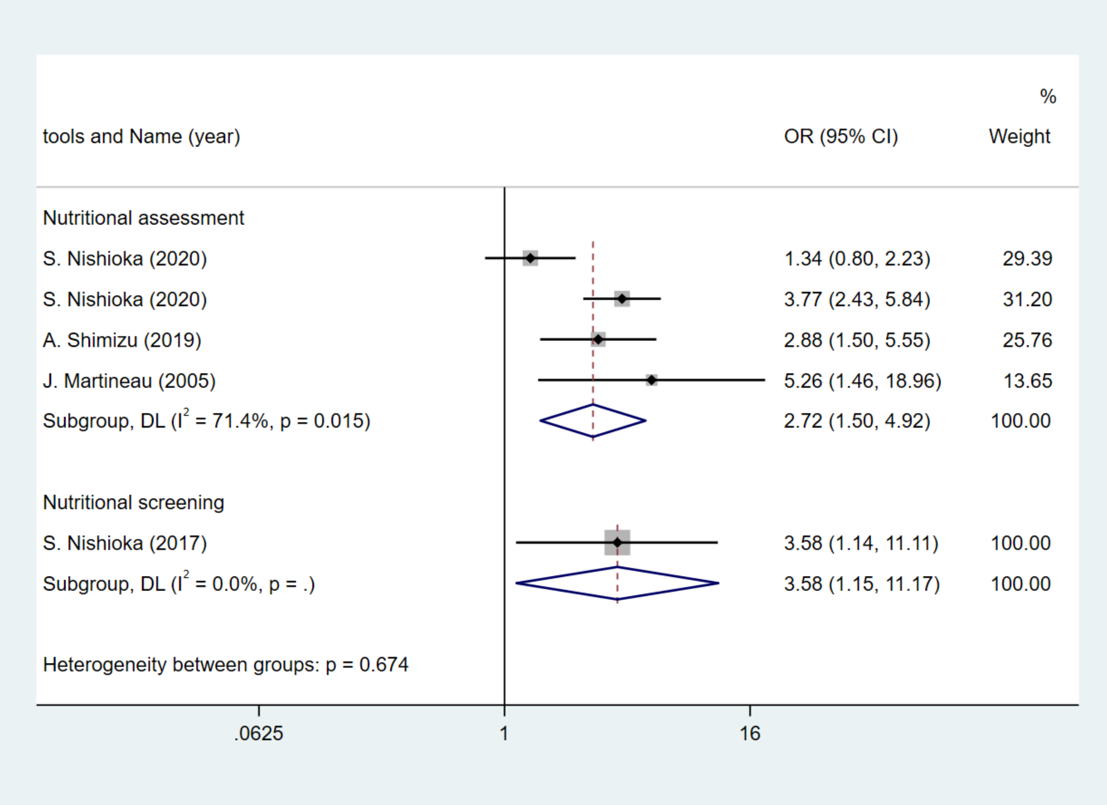

Figure S6. Subgroup analysis of the impact of malnutrition identified by each tool on dysphagia in patients with stroke.

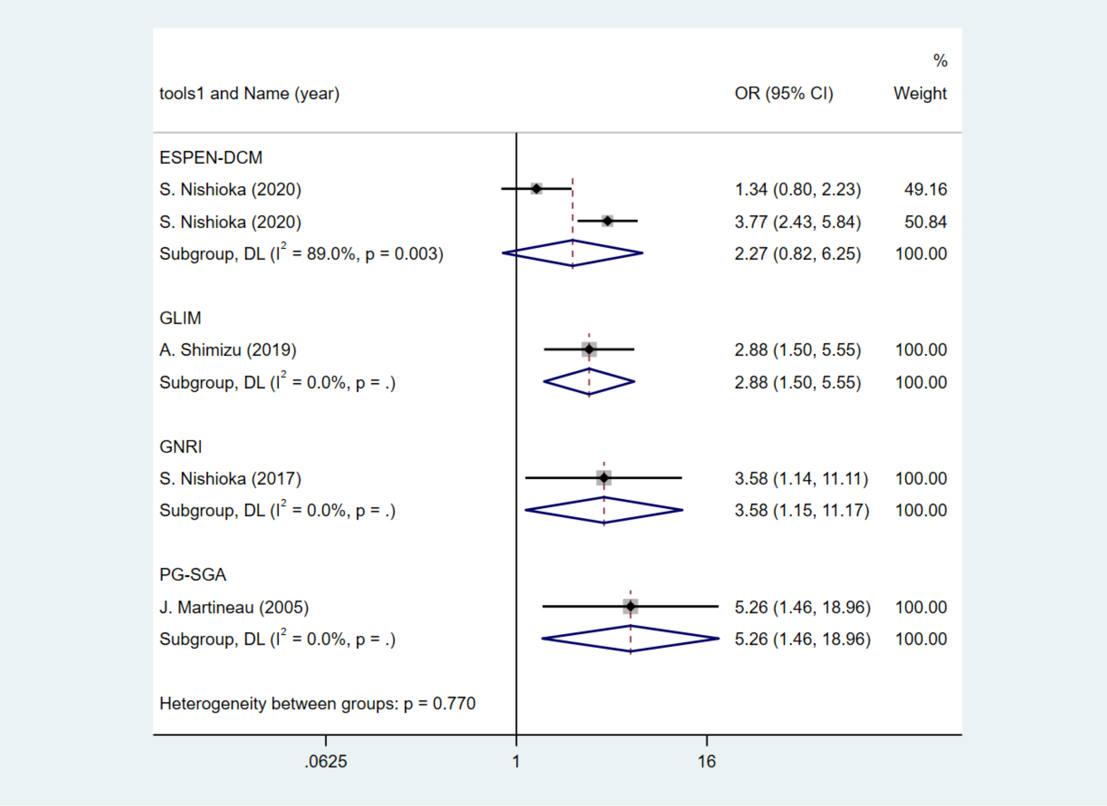

Figure S7. Subgroup analysis of the impact of malnutrition identified by each tool on poor functional outcomes in patients with stroke.

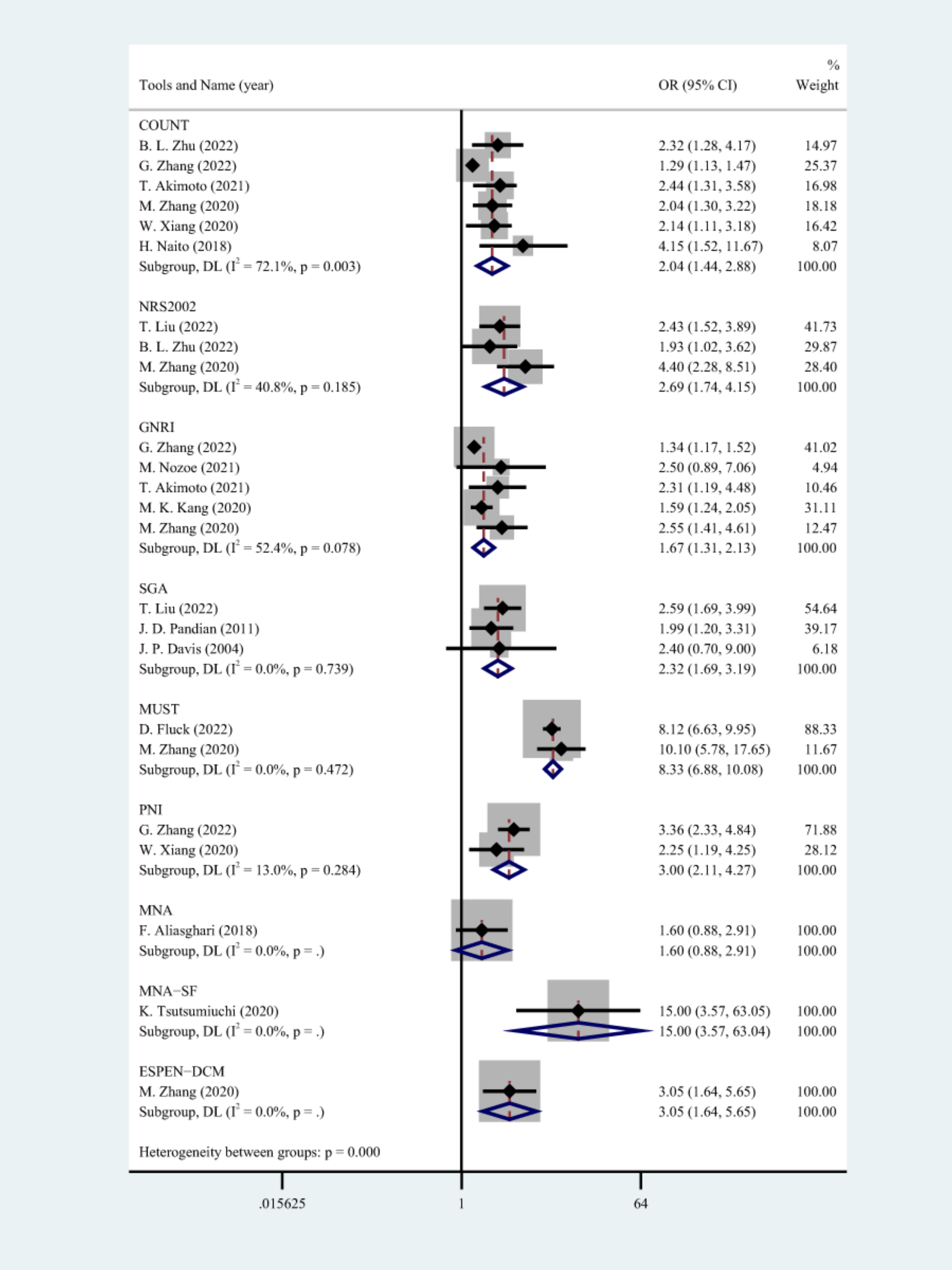

Figure S8: Funnel plot

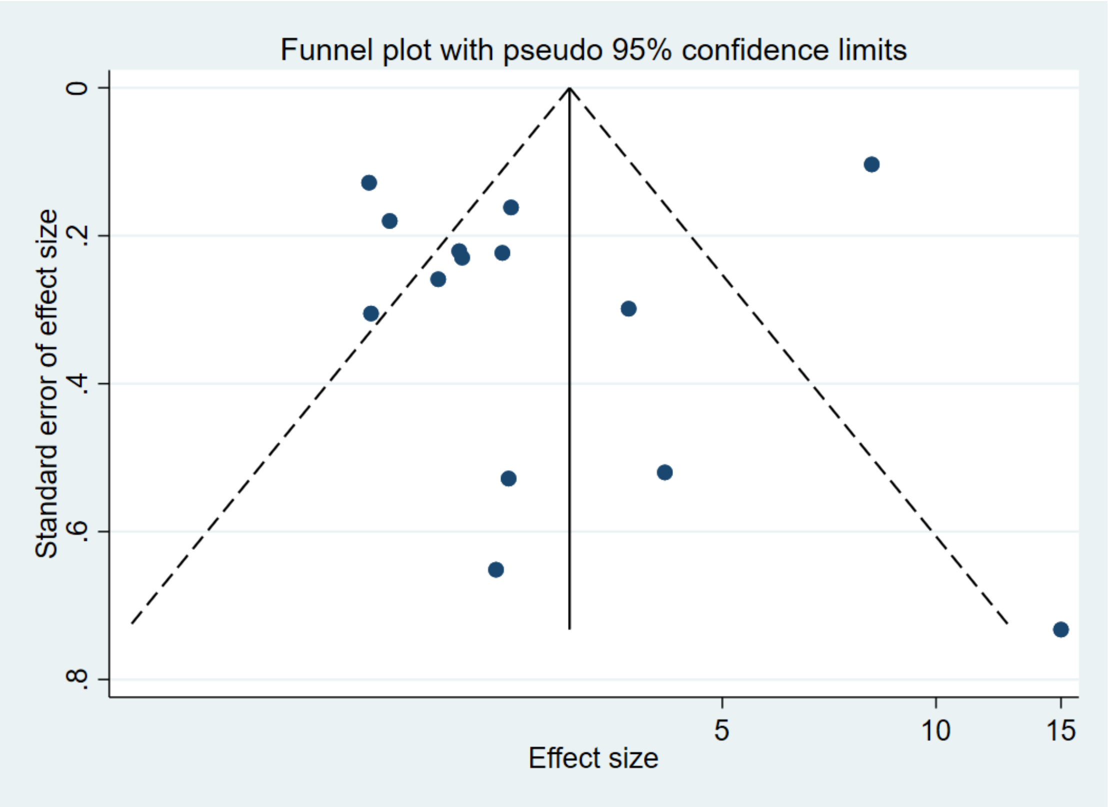

Table S1. Risk of bias assessment by ROBINS-I.

Study	Bias due to confounding	Bias in selection of participants into the study	Bias in measurement classification of interventions	Bias due to deviations from intended interventions	Bias due to missing data	Bias in measurement of outcomes	Bias in selection of the reported result	Overall
Zhu 2022	Moderate	Low	Low	Moderate	Moderate	Low	Moderate	Moderate
Sato 2022	Moderate	Moderate	Low	Moderate	Moderate	Low	Moderate	Moderate
Liu 2022	Moderate	Serious	Low	Moderate	Moderate	Low	Moderate	Serious
Lee 2022	Moderate	Moderate	Low	Moderate	Serious	Low	Moderate	Serious
Kamimoto 2022	Serious	Moderate	Low	Moderate	Low	Low	Serious	Serious
Fluck 2022	Moderate	Low	Low	Moderate	Low	Low	Moderate	Moderate
Zhang 2022	Moderate	Low	Low	Moderate	Low	Low	Moderate	Moderate
Nozoe	Moderate	Low	Low	Moderate	Moderate	Low	Moderate	Moderate
Akimoto 2021	Serious	Moderate	Low	Moderate	Moderate	Low	Serious	Serious
Scrutinio 2020	Moderate	Low	Low	Moderate	Moderate	Low	Moderate	Moderate
Nishioka 2020	Moderate	Moderate	Low	Serious	Serious	Low	Moderate	Serious
Nishioka 2020	Serious	Low	Low	Moderate	Moderate	Low	Moderate	Serious
Kokura 2020	Serious	Moderate	Low	Moderate	Moderate	Low	Moderate	Serious
Irisawa 2020	Serious	Low	Low	Moderate	Low	Low	Moderate	Serious
Kang 2020	Low	Low	Low	Low	Low	Low	Moderate	Moderate
Zhang 2020	Moderate	Low	Low	Moderate	Moderate	Low	Moderate	Moderate
Xiang	Moderate	Low	Low	Moderate	Low	Low	Moderate	Moderate
Tsutsumiuchi 2020	Moderate	Moderate	Low	Moderate	Moderate	Low	Moderate	Moderate
Shimizu 2019	Moderate	Low	Low	Moderate	Moderate	Low	Moderate	Moderate

Naito 2018	Serious	Moderate	Low	Moderate	Moderate	Low	Moderate	Serious
Aliasghari 2018	Moderate	Serious	Low	Moderate	Low	Low	Moderate	Serious
Nishioka 2017	Low	Low	Low	Moderate	Low	Low	Low	Moderate
Kokura 2016	Moderate	Moderate	Low	Moderate	Moderate	Low	Moderate	Moderate
Pandian 2011	Moderate	Low	Low	Moderate	Low	Low	Moderate	Moderate
Martineau 2005	Serious	Low	Low	Moderate	Moderate	Low	Serious	Serious
Davis 2004	Moderate	Low	Low	Moderate	Moderate	Low	Moderate	Moderate

Abbreviations: NRSIs, non-randomized studies of interventions (including cohort studies, case-control studies, etc.); ROBIN-I, risk of bias in non-randomized studies of interventions.
